# Methyltransferase as Antibiotics Against Foodborne Pathogens: An *In Silico* Approach for Exploring Enzyme as Enzymobiotics

**DOI:** 10.3389/fgene.2021.800587

**Published:** 2022-01-03

**Authors:** Varish Ahmad, Aftab Ahmad, Mohammed F. Abuzinadah, Salwa Al-Thawdi, Ghazala Yunus

**Affiliations:** ^1^ Health Information Technology Department, Faculty of Applied Studies, King Abdulaziz University, Jeddah, Saudi Arabia; ^2^ Department of Medical Laboratory Technology, Faculty of Applied Medical Sciences, King Abdulaziz University, Jeddah, Saudi Arabia; ^3^ Department of Biology, College of Science, University of Bahrain, Sakhir, Bahrain; ^4^ Department of Basic Science, University of Hail, Hail, Saudi Arabia

**Keywords:** methyltransferase, antimicrobial, drug resistant, protein–protein interaction, enzymobiotics

## Abstract

The development of resistance in microbes against antibiotics and limited choice for the use of chemical preservatives in food lead the urgent need to search for an alternative to antibiotics. The enzymes are catalytic proteins that catalyze digestion of bacterial cell walls and protein requirements for the survival of the cell. To study methyltransferase as antibiotics against foodborne pathogen, the methyltransferase enzyme sequence was modeled and its interactions were analyzed against a membrane protein of the gram-positive and gram-negative bacteria through *in silico* protein–protein interactions. The methyltransferase interaction with cellular protein was found to be maximum, due to the maximum PatchDock Score (15808), which was followed by colicin (12864) and amoxicillin (4122). The modeled protein has found to be interact more significantly to inhibit the indicator bacteria than the tested antibiotics and antimicrobial colicin protein. Thus, model enzyme methyltransferase could be used as enzymobiotics. Moreover, peptide sequences similar to this enzyme sequence need to be designed and evaluated against the microbial pathogen.

## Introduction

In fact, chemotherapy has renovated the treatments not only against bacterial disease but also fungal diseases. However, many pathogens become protective against available antibiotics and pose a threat to the health of humans and animals. Various alternatives to antibiotics such as probiotics, nanobiotics, antimicrobial peptides or bacteriocin, CRISPR-Cas, quorum-sensing inhibitors, phage therapy, and immunotherapy exist (Kumar et al., 2021).

The enzymes are proteinaceous molecules and known as biocatalysts or endopeptidases. Recent research has reported that enzymes could be used as a special class of antimicrobial enzymobiotics, against microbial infections and to control the drug-resistant microbes.

Enzymes play a significant role in the expression of cellular proteins, cell wall polysaccharides, nucleic acids, and other cellular metabolites that are required for the survival of the cell. The use of enzymes as bacteriophage holins and their membrane-disrupting activity, anti-staphylococcal lytic enzymes, and membrane-targeted antibiotics have been recently highlighted by much research.

Enzymes like glucose oxidase, hydrogen peroxidase, and protease have inhibitory effects on microbial pathogens. The bacteriostatic and inhibitory effect on biofilm formation against many pathogenic bacteria like *P. aeruginosa*, *S. aureus*, and methicillin-resistant *S. aureus*, with a patented formulation of glucose oxidase, was recently explored by Cooper (2013). The glucose oxidase reported inhibiting *Staphylococcus* cells more potent than the *P. aeruginosa* cells (Cooper, 2013). Bacterial lipopolysaccharides (LPS) are involved in maintaining intestinal homeostasis and mediate potent pro-inflammatory toxins/mediators. Thus, apical brush borders rich in alkaline phosphatase are analyzed as a significant de-phosphorylation molecule for the neutralization of LPS in addition to un-methylated cytosine-guanosine dinucleotides and flagellin, resulting in reduced toxicity and inflammatory responses (Drago-Serrano et al., 2012).

A recent study was conducted to investigate the effect of dietary proteases on nutrient digestibility, growth performance, crude protein digestibility, enzyme (pepsin, pancreatic amylase, and trypsin) activities, plasma total proteins, intestinal villus heights, intestinal morphology, and the expression levels of specific genes. Significant increases in growth performance have been observed which were attributed to better intestinal development, enhanced protein digestibility, and improved nutrient transport efficiencies. The supplementation of proteases (200 and 300 mg/kg) within the diet increased the ratio of villus heights to crypt depth significantly, especially in the duodenum, jejunum, and ileum, and induced higher expression levels of the peptide transporter 1 (PepT1) within the duodenum region (Zuo et al., 2015).

The microorganisms are very sensitive to utilizing the nutrients from crops through microbial enzymatic actions. The main enzymes that help to initiate the deterioration are the first attacker on the cell wall, and they are popularly known as cell wall degrading enzymes. The cell wall degrading enzymes could be employed as antibiotics. Many proteinaceous molecules like bacteriocin produced from plants, animals, and microbes have been tested as potential therapeutic molecules. Initially, a homogeneous microbial population has grown and started the deterioration that is further exposed to the new environment to favor the growth of other pathogens. This resulted in the growth of heterogeneous microbial populations on the same habitats to initiate spoilage or pathogenesis by damaging the cellular components, thus helping tissue attack and microbial dissemination (Kikot et al., 2009). To inhibit the growth of these foodborne pathogens, chemical preservatives are not the preferred choice for food (Chukwuka et al., 2010).

Moreover, the microbial resistance against currently used antibiotics has raised serious human and animal health issues globally. This antimicrobial-resistant is well reported in many microbes including bacteria and fungi against last-line antibiotics, signifying a future loss of the therapeutic option to treat the infections. Many scientific strategies have also been tested to combat the drug-resistant microbes (Sartelli et al., 2017). The developed countries and developing countries have many challenges that can spread and stimulate the emergence of multidrug-resistant pathogen among microbial populations. The drug-resistant bacteria like *Pseudomonas aeruginosa* (*P. aeruginosa*), *K. pneumonia*, *Streptococcus pneumoniae*, and *Staphylococcus aureus* have been well reported and recognized as a global threat (Ventola, 2015). Managing these challenges need many scientific efforts that explore microbial resistance and the designing of effective controlling strategies such as active surveillance that stop the development and spread of drug-resistant microbes in the country. Moreover, improper use of antibiotics, infection inhibition, and control safeguards should also be improved to limit further spread. Therefore, it is highly important to explore the alternatives to antibiotics, such as the use of antimicrobial peptides, bacitracin, or lactic acid bacteria must be promoted for the primary control of microbes. Therefore, this *in silico* based study explores the use of the enzyme methyltransferase sequence as antibiotics against gram-negative and gram-positive bacteria.

## Materials and Methods

### Preparation of Ligand Molecules

**FIGURE 1:**
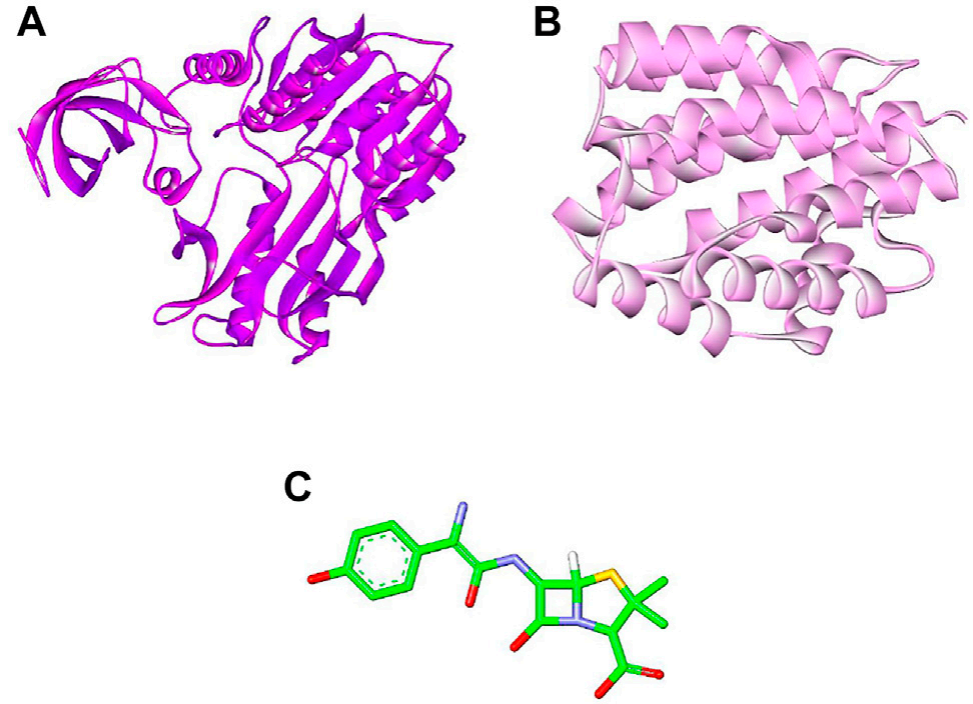
Selected molecules as ligand **(A)** methyltransferase (purple color), **(B)** colicin protein (light pink color), and **(C)** amoxicillin (green color).

The structural information of amoxicillin (AMX) was retrieved from the DrugBank database (https://go.drugbank.com/drugs/DB01060) (Wishart et al., 2008) (Figure 1C). The 3D structure of 23S rRNA [uracil (1939)-C (5)]-methyltransferase RlmD (*Pediococcus acidilactici*) (Sequence ID: WP_004165491.1) sequence was obtained from the National Centre for Biotechnology Information (NCBI) and modeled using the SWISS MODEL server (Waterhouse et al., 2018) (Figure 1A). The colicin structure was downloaded from the Protein Data Bank (RCSB PDB - 1COL: refined structure of the pore-forming domain of colicin a at 2.4 angstroms resolution) (Figure 1B).

### Preparation of Receptor Molecules (SWISS-MODEL Workspace/GMQE)

**FIGURE 2:**
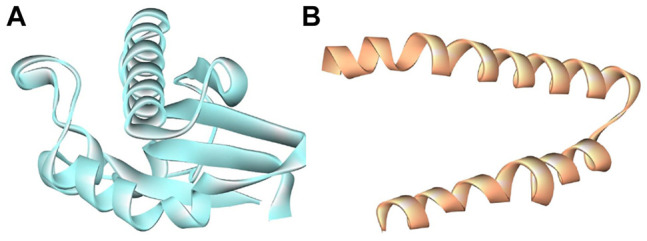
Selected biomolecules as receptor **(A)** *E. coli* membrane protein (light turquoise color) and **(B)** *Staphylococcus aureus* membrane protein (golden color).

We have accessed the PDB database for the receptor molecules but did not find them. Therefore, the 3D structures of membrane protein (*Escherichia coli*) (accession no.: APJ97041.1) (Figure 2A) and membrane protein (*Staphylococcus aureus*) (accession no.: KII21430.1) (Figure 2B) were modeled after retrieving their sequences in the FASTA format from the NCBI and provided as an input for the SWISS MODEL server on the basis of homology approaches (detailed information available in Supplementary Materials).

### Model Evaluation

All modeled 3D structures were evaluated using the MolProbity version 4.4 assessment tool integrated in the SWISS-MODEL server (Chen et al., 2010). All-atom structure validation for macromolecular crystallography was carried out.

### Molecular Docking

The molecular interaction analysis were executed using the PatchDock online server (https://bioinfo3d.cs.tau.ac.il/PatchDock/) (Duhovny et al., 2002; Schneidman-Duhovny et al., 2005). PatchDock uses a geometry-based molecular docking algorithm as a scoring function. All figures were generated using Discovery Studio Visualizer 2020 (Ventola, 2015; Dassault Systèmes, 2020).

### MDS Experimentation

The docking results *E. coli*_mem, *E. coli*_mem + COL complexes, and *E. coli*_mem + MT of complexes were further analyzed by MDS studies using advanced computational techniques. Thus, the MDS environment was created, and simulation study was conducted for 50 nanoseconds (ns) using the GROningen MAchine for Chemical Simulations (GROMACS) tool (2018 version) (Van Der Spoel et al., 2005) developed by the University of Groningen, Netherlands. The simulation in water for complexes *E. coli*_mem, *E. coli*_mem + MT, and *E. coli*_mem + COL was performed by using GROMACS standard protocol.

The simulation for selected complexes, initially, the pdb2gmx module, was utilized and required *E. coli*_mem, *E. coli*_mem + MT, and *E. coli*_mem + COL topology files to be generated, followed by OPLS-AA/L all-atom force field selection.

The solvation step was performed by creating a unit water willed cell cubic box. The energy was minimized by addition of Na+ and Cl- ions for stabilization of the system. Equilibrium setup for the (all complexes) system was essential and created, followed by two-step ensembles NVT and NPT (constant N, number of particles; V, volume; P, pressure; T, temperature) providing constancy and stabilization of the system through complete simulation (Gupta et al., 2020).

GORMACS have many packages, for *E. coli*_mem, *E. coli*_mem + MT, and *E. coli*_mem + COL complexes. MDS analysis, root mean square deviation (RMSD) was analyzed by gmx rms (Kufareva and Abagyan, 2012), root mean square fluctuation (RMSF) was analyzed by gmx rmsf for, gmx gyrate for the calculation of radius of gyration (Rg) (Kuzmanic and Zagrovic, 2010), and gmx. Finally, after a successful 50-ns simulation run, trajectory files and graphical plots were generated by using the xmgrace program (Turner, 2005).

## Results

**FIGURE 3:**
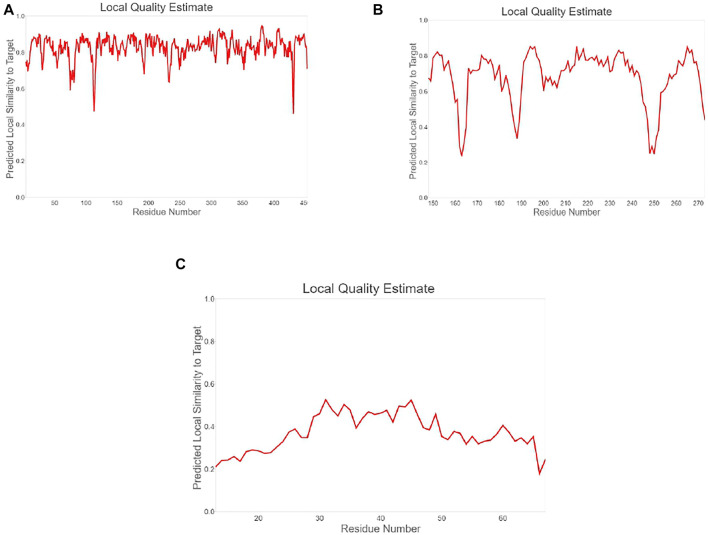
Plot showing local quality estimation of **(A)** methyltransferase, **(B)** *E. coli* membrane protein, and **(C)** *Staphylococcus* membrane protein.

**FIGURE 4:**
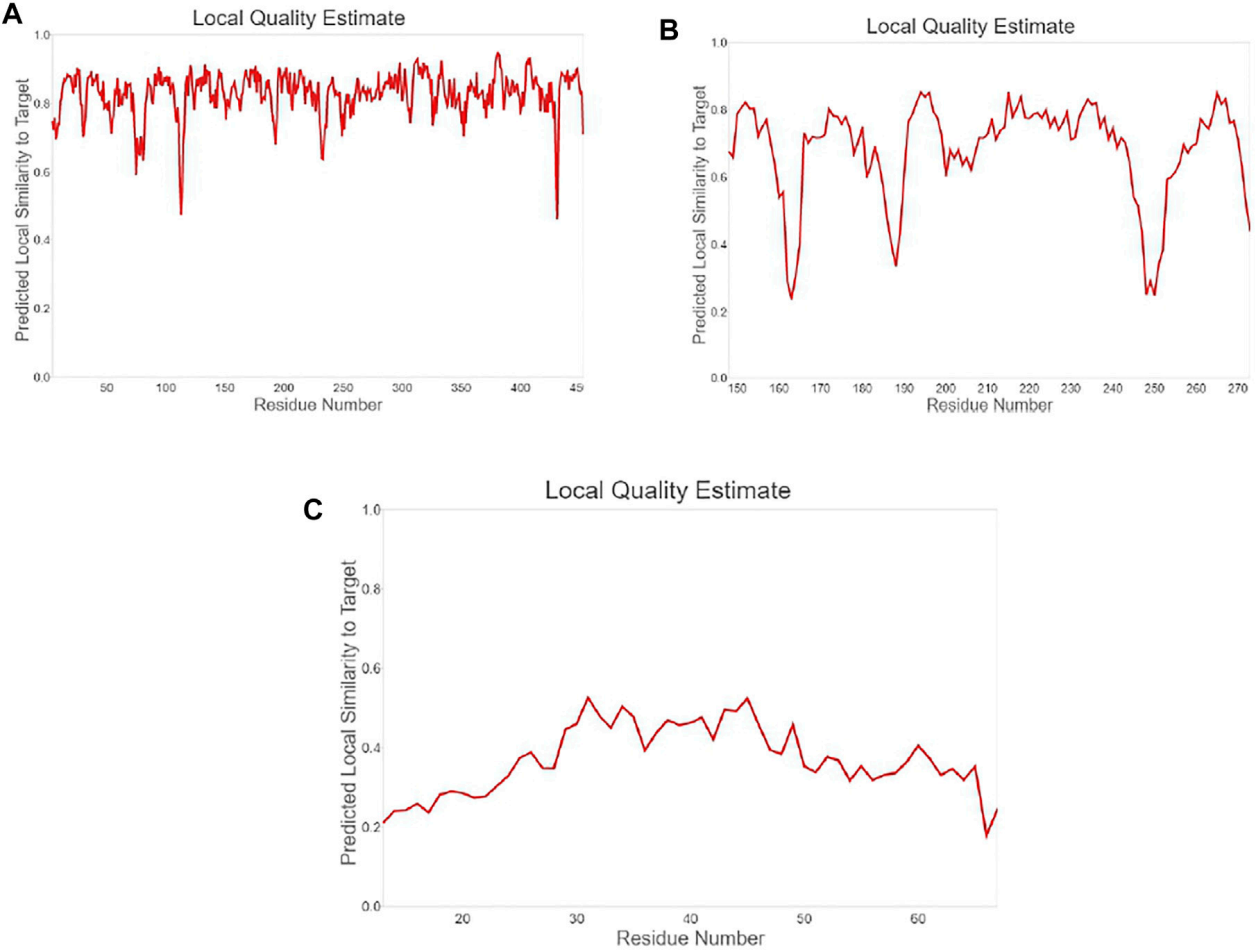
Plot showing local quality estimation of **(A)** methyltransferase, **(B)** *E. coli* membrane protein, and **(C)** *Staphylococcus* membrane protein.

To study the binding interaction of methyltransferase with the cellular protein of gram-positive and gram-negative bacterial pathogens, the ligand and receptor structure was created using SWISS-MODEL (Figures 1A–C, 2A–C). Similarly, the ligand 3D structure of membrane protein (*Escherichia coli*) and (accession no.: APJ97041.1) and membrane protein (*Staphylococcus aureus*) (accession no.: KII21430.1) was modeled after retrieving their sequences in the FASTA format from the NCBI and provided as an input for the SWISS-MODEL server on the basis of homology approaches. All modeled 3D structures were evaluated using the MolProbity version 4.4 assessment tool integrated into the SWISS-MODEL server. The model structure information is represented in Table 1, and the local quality of models is represented in Figures 3, 4.

**TABLE 1:** Showing quality assessment results of modeled 3D structures.

Model	MolProbity score	Clash score	Ramachandran favored	Ramachandran outliers	Rotamer outliers	C-Beta deviations
Ideal case	As low as possible	0	>98%	<0.2%	<1%	0
Methyltransferase	1.04	0.70	95.55%	1.11%	0.51%	1
Membrane protein (*Escherichia coli*)	1.77	4.56	95.16%	0.81%	1.83%	2
Membrane protein (*Staphylococcus aureus*)	0.50	0.00	98.11%	0.00%	0.00%	0

**FIGURE 5:**
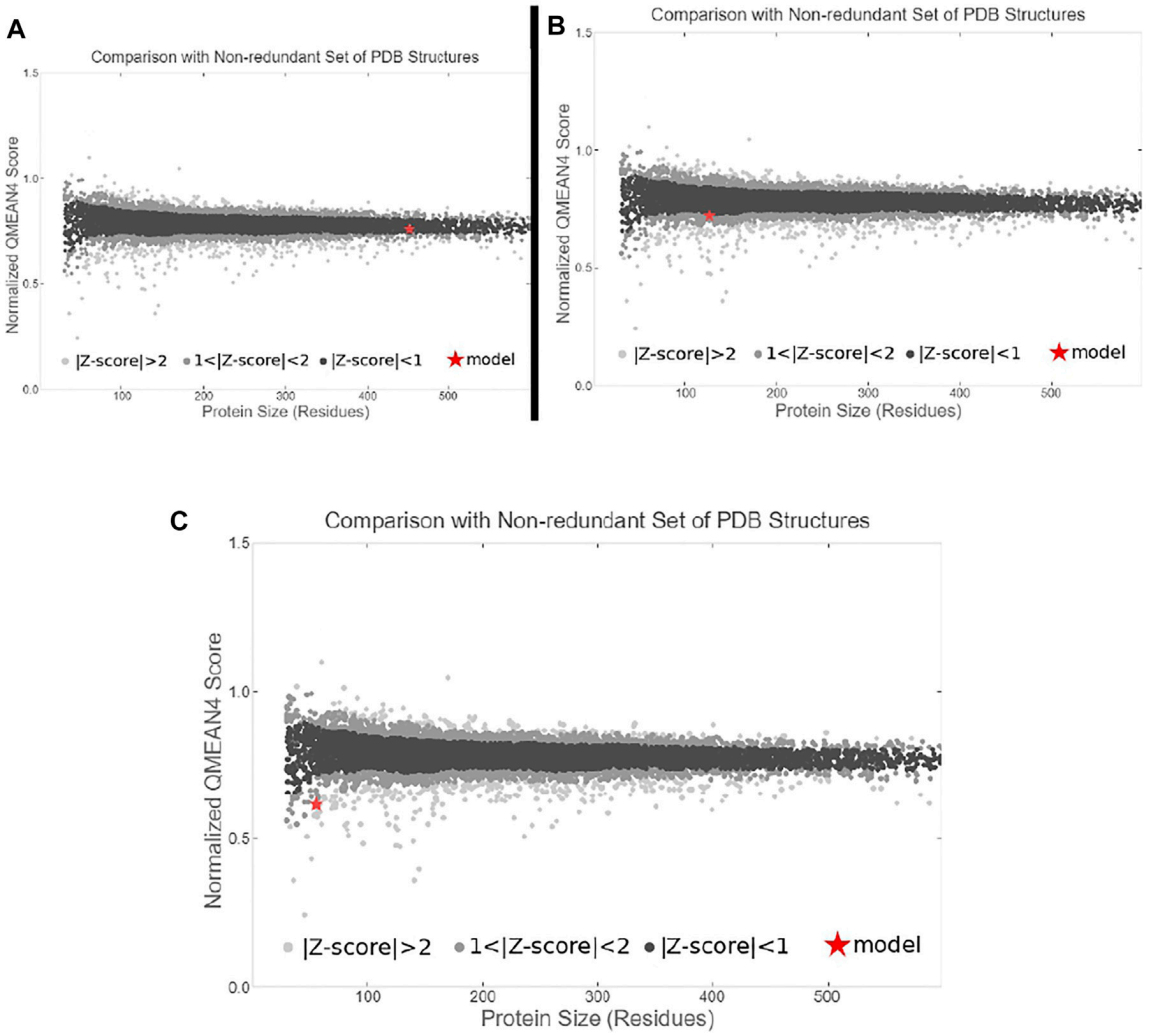
Plot showing local quality comparison with non-redundant set of PDB structure **(A)** methyltransferase, **(B)** *E. coli* membrane protein, and **(C)** *Staphylococcus* membrane protein.

**FIGURE 6:**
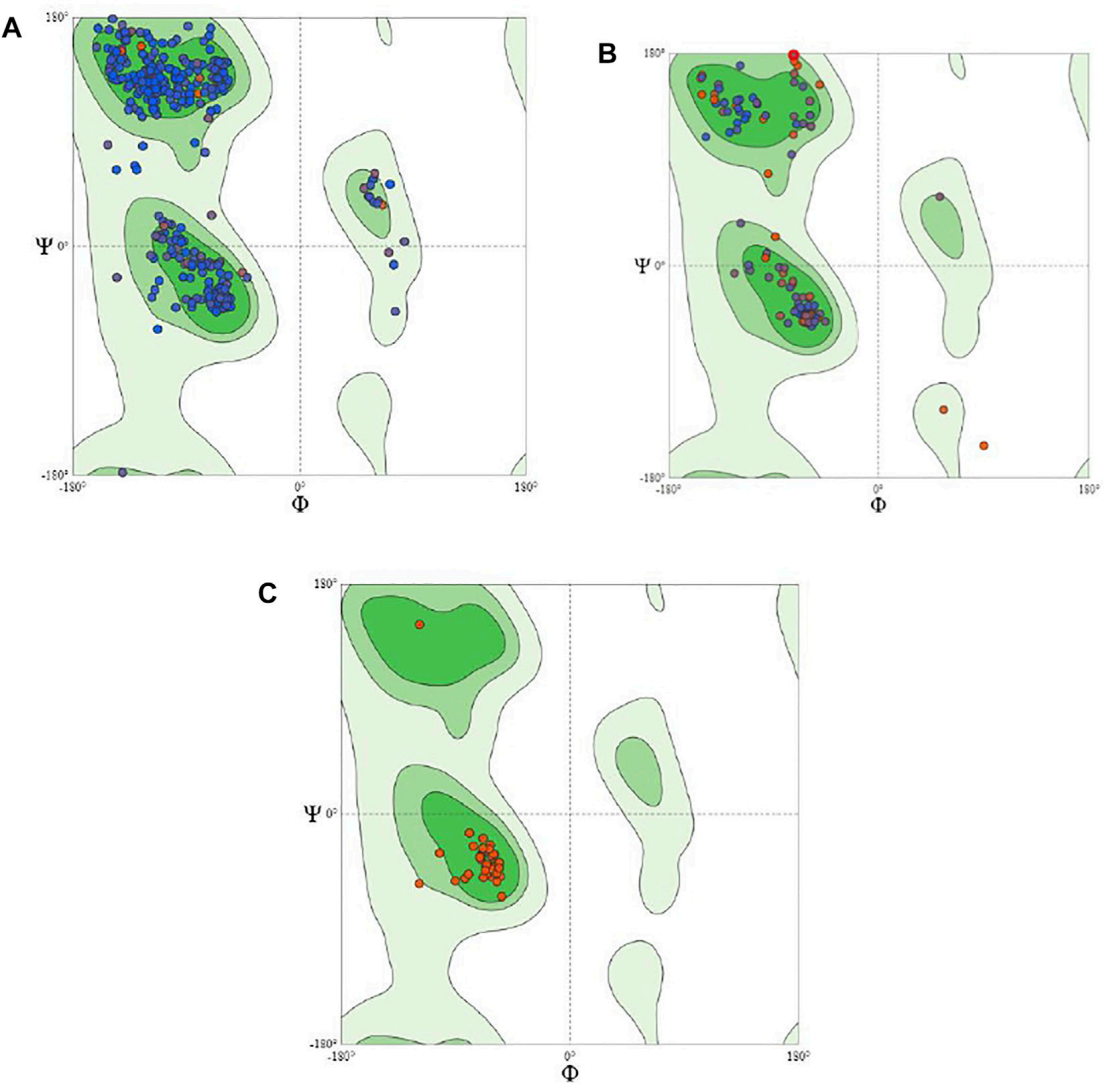
Showing Ramachandran plot for modeled 3D structures of structures **(A)** methyltransferase, **(B)** *E. coli* membrane protein, and **(C)** *Staphylococcus* membrane protein.

**FIGURE 7:**
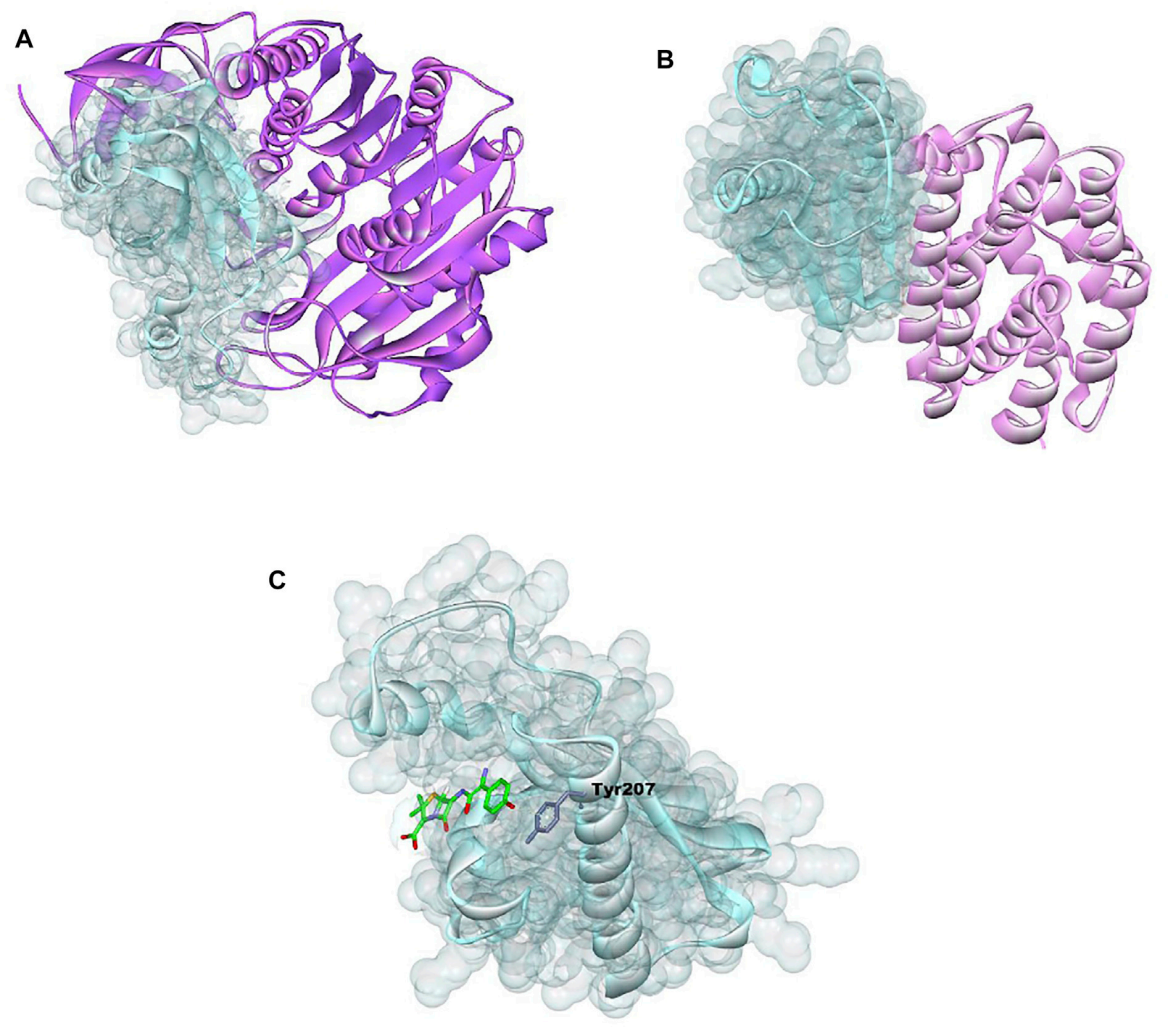
Showing 3D visualization of *E. coli* membrane protein (shown in light turquoise color with solid ribbon pattern) interaction with **(A)** methyltransferase (purple color in ribbon pattern), **(B)** colicin protein (light pink color in ribbon pattern), and **(C)** amoxicillin (green color in stick pattern).

The quality comparison with a non-redundant set of PDB structures is also performed, which is shown in Figure 5. The stability of modeled ligand molecules and receptors was confirmed by the Ramachandran plot (Figure 6), which shows that the modeled 3D structure of membrane protein (*Staphylococcus aureus*) was the best-predicted structure that had 98.11% amino acid residues in the favored region with no C-Beta deviation (Table 1 and Figure 7A–C).

Furthermore, the molecular interaction analysis was executed using the PatchDock online server (https://bioinfo3d.cs.tau.ac.il/PatchDock/) (Duhovny et al., 2002; Schneidman-Duhovny et al., 2005; Ansari et al., 2020) (Tables 2 and 3).

**TABLE 2:** *E. coli* membrane protein interaction with methyltransferase, colicin protein, and amoxicillin. In the hydrogen bond column, EC, *E. coli* membrane protein; MT, methyltransferase; COL, colicin protein; AMX, amoxicillin.

Serial number	Ligand molecule	PatchDock score	Hydrogen bonds	Hydrogen bonds length (Angstrom)
1	Methyltransferase	15808	EC:LYS163:N—MT:GLY153:O	2.91143
			EC:LYS240:NZ—MT:THR251:OG1	3.15602
			MT:SER111:OG—EC:GLU217:OE1	2.58874
			MT:ASN252:N—EC:ARG201:O	2.39953
			MT:ARG387:NH2—EC:ASP206:O	1.93498
			MT:ARG387:NH2—EC:THR210:OG1	2.16363
			EC:LYS163:CA—MT:ARG152:O	3.48398
			MT:ARG418:CD—EC:SER235:O	2.69943
			MT:HIS445:CE1—EC:THR210:OG1	3.19989
2	Colicin protein	12864	EC:ARG150:NH1—COL:ASP24:OD2	2.88023
			EC:THR152:OG1—COL:GLU17:OE2	3.36734
			EC:LYS245:NZ—COL:LYS6:O	2.9338
			EC:LYS246:NZ—COL:ASN47:OD1	3.30611
			COL:LYS6:NZ—EC:SER255:O	3.01983
			EC:ARG150:CD—COL:GLU17:O	2.74897
			COL:LYS97:CE—EC:HIS179:O	2.22174
			COL:GLY166:CA—EC:GLY274:OXT	3.08932
3	Amoxicillin	4122	AMX:O—EC:TYR207	3.93411

**TABLE 3:** *Staphylococcus* membrane protein interaction with methyltransferase, colicin protein, and amoxicillin. In the hydrogen bond column, STP, *Staphylococcus* membrane protein; MT, methyltransferase; COL, colicin protein; AMX, amoxicillin.

Serial number	Ligand molecule	PatchDock score	Hydrogen bonds	Hydrogen bonds length (Angstrom)
1	Methyltransferase	14024	STP:LYS36:NZ—MT:GLN286:OE1	3.12548
			STP:GLN42:NE2—MT:SER154:O	3.36677
			STP:LYS45:NZ—MT:LYS151:O	3.28368
			STP:LYS45:NZ—MT:ARG152:O	2.83486
			MT:GLN135:NE2—STP:MET34:SD	3.7311
			MT:SER154:N—STP:GLN42:OE1	2.89249
			MT:ASN252:ND2—STP:LYS45:O	2.9043
			STP:HIS37:CE1—MT:GLN166:OE1	3.75744
			MT:HIS445:CE1—STP:VAL35:O	2.55974
2	Colicin protein	12790	COL:PRO132:CD—STP:ASP26:OD1	2.24668
3	Amoxicillin	3544	STP:ARG58:NH1—AMX:O	1.67897
			AMX:O—STP:LEU51:O	3.26094
			AMX:N—STP:PHE55	3.93687

**FIGURE 8:**
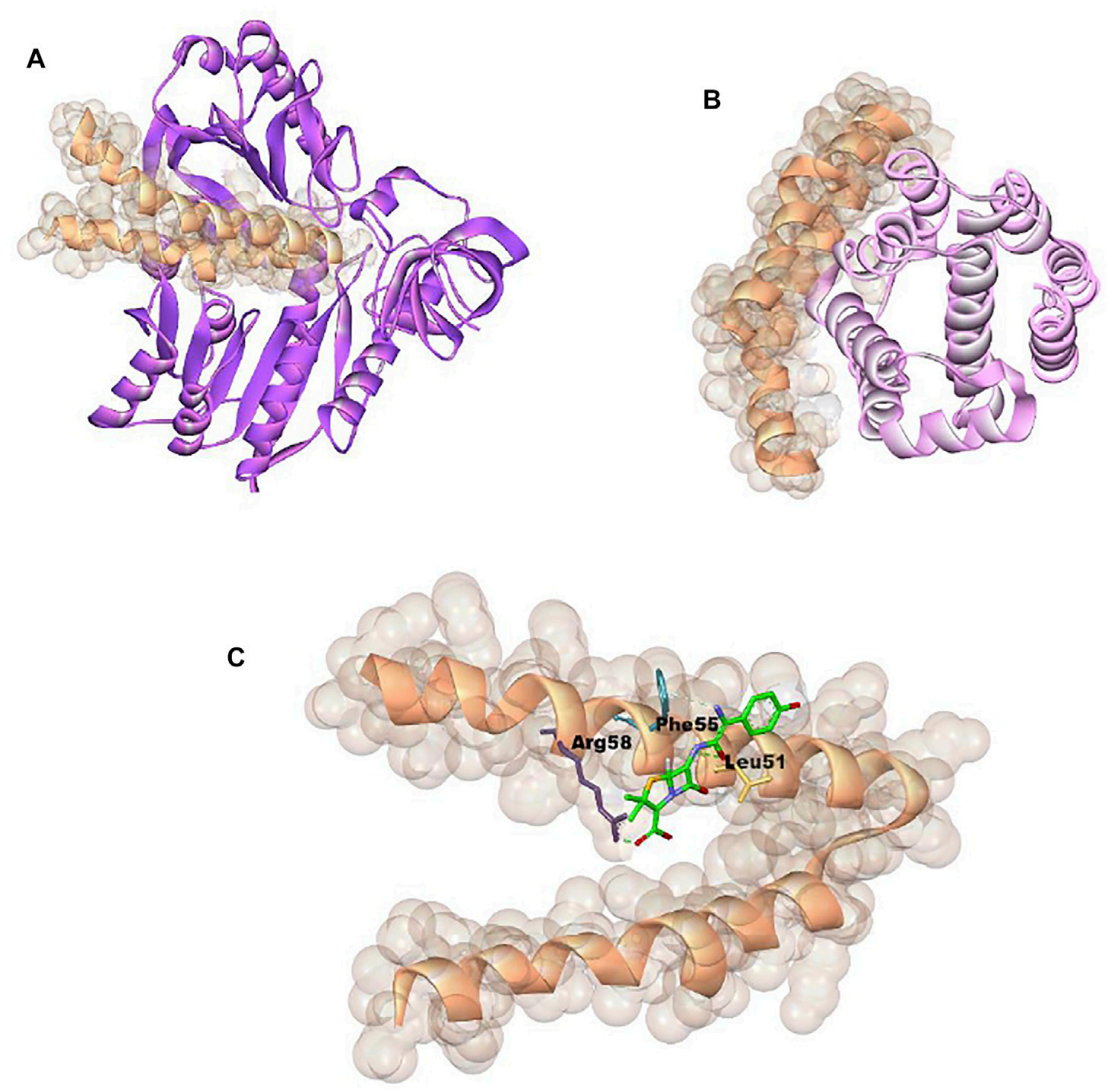
Showing 3D visualization of *Staphylococcus aureus* membrane protein (shown in golden color with solid ribbon pattern) interaction with **(A)** methyltransferase (purple color in ribbon pattern), **(B)** colicin protein (light pink color in ribbon pattern), and **(C)** amoxicillin (green color in stick pattern).

*E. coli* membrane protein interaction with methyl transferase with colicin protein and amoxicillin were analyzed and 3D graphics generated using Discovery Studio Visualizer 2020 (Figure 8).

The methyltransferase interaction with cellular protein was found to be maximum, due to the maximum PatchDock Score (15808), which was followed by colicin (12864) and amoxicillin (4122) (Table 2; Figures 7A–C). Moreover, the interaction bond was stabilized through the hydrogen bond between methyltransferase, colicin, amoxicillin, and cellular protein; EC: LYS163:N—MT:GLY153:O, EC:ARG150:NH1—COL:ASP24:OD2, and AMX:O—EC:TYR207. Similarly, interaction study with methytransferase, colicin, and amoxicillin with cellular protein of bacterial pathogen *S. aureus* were found to the maximum with methyltransferase (PatchDock Score: 14024), followed by colicin (PatchDock Score 12790) and amoxicillin (Table 3; Figures 8A–C).

### MDS Analysis

Furthermore, after the MDS total experimentation 50 ns run, the analysis was done on the basis of obtained data from RMSD, RMSF, and Rg plot analysis, revealing deviation, fluctuation, and stability of *E. coli_*mem, *E. coli*_mem + MT, and *E. coli*_mem + COL complexes during the whole simulation period. The RMSD values for selected simulated molecules ranged between 0.15 and 0.4 nm (Figure 7A). The observed RMSD values for *E. coli* _mem, *E. coli*_ mem + MT, and *E. coli*_mem + COL complexes were between 0.2 and 0.25 nm, 0.3–0.4 nm, and 0.2–0.25 nm, respectively. In comparison with *E. coli*_mem, the *E. coli*_mem + MT, and *E. coli*_mem + COL complexes showed stability after 20 ns until 50 ns (Figure 7A). The *E. coli*_mem + MT complex average RMSD value was higher than those of the *E. coli*_mem and *E. coli*_mem + COL complexes.

**FIGURE 9:**
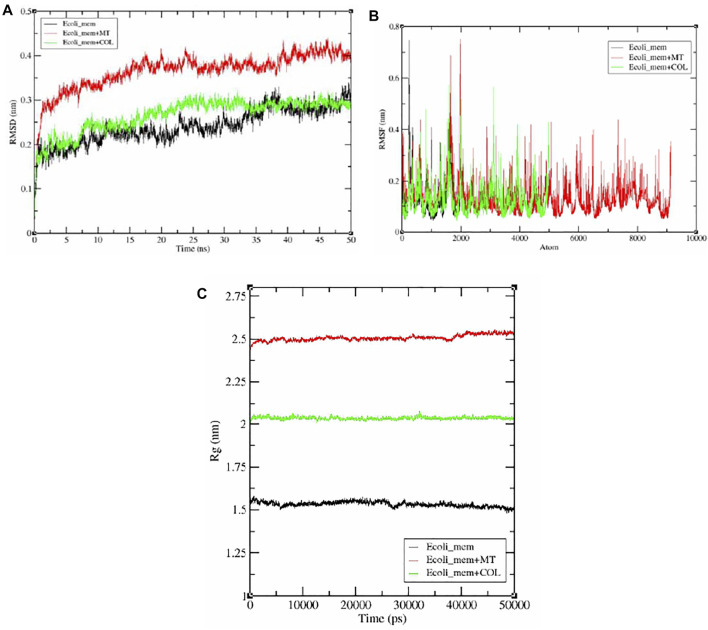
Graphical representation **(A)** RMSD plot of *E. coli*_mem (black color), *E. coli*_mem + MT (red color), and *E. coli*_mem + COL (green color) and showing deviation and stability during 100-ns period. **(B)** RMSF plot with fluctuation per residues. **(C)** Radius of gyration (Rg) plot showing compactness of *E. coli*_mem, *E. coli*_mem + MT, and *E. coli*_mem + COL molecule during 50000ps simulation. Where nm, nanometer; ns, nanosecond; ps, picosecond.

RMSF calculation per atom showed a value that ranged between 0.1 and 0.7 nm for protease, *E. coli*_mem, *E. coli*_mem + MT, and *E coli*_mem + COL complexes, and it was observed that for most of the residues, the RMSF value remains near 0.1 nm for all complexes (Figure 9A–C). Furthermore, few fluctuations were observed at the 2000- and 3000-atom regions. (Figure 9B).

Radius of gyration (Rg) analysis is very important for the assessment of the compactness and stability of the protein structure during the whole simulation period. The *E. coli*_mem Rg plot shows an average value of approximately 1.5 nm. *E. coli*_mem + MT and *E. coli*_mem + COL remain stable and show average values near 2.0 and 2.25 nm, respectively. No major fluctuation was observed in Rg plot analysis (Figure 9C).

## Discussion

Currently, a formulation of glucose oxidase with other active ingredients was patented. The formulation has been described to has the inhibitory potential against foodborne bacteria *Staphylococcus* cells and biofilm formation of *P. aeruginosa, S. aureus*, and methicillin-resistant *S. aureus*. Honey is rich in nutrient value, glucose oxidase which inhibits *P. aeruginosa*.

Moreover, breeding of novel honeybee species that have produced more glucose oxidases in order to increase the antibacterial efficacy of the product (Bucekova et al., 2014).

The enzymes like endopeptidases themselves are required for the normal growth of bacteria. Moreover, it also destroyed the bacterial cell well in presence of beta-lactam antibiotics like penicillin (Shin et al., 2016). The antimicrobial potential of many ribosomally synthesized proteins named bacteriocin has been reported to kill or inhibit the growth of gram-positive and gram-negative bacteria (Ahmad et al., 2014; Ahmad et al., 2019). In this study, the protein–protein interaction study was conducted between the methyltransferase and cellular membrane protein of gram-positive bacteria and gram-negative bacteria. The antibiotics amoxicillin and peptide antibiotic colicin, a well-known antimicrobial peptide were studied as a positive control. Colicin is the first potential antimicrobial peptide produced from *E. coli* bacteria that has a bactericidal effect by forming pores in the inner membrane of nonhost *E. coli* and damaging the DNA and RNA (Cascales et al., 2007; Jin et al., 2018). The enterococin A, the protein–protein interactions of methyltransferase with cell protein of *E. coli* was observed to be more significant than that of the colicin and amoxicillin as it has been observed to be the maximum PatchDock score (15808), which is followed by colicin (12864) and amoxicillin (4122). The interaction of cellular protein was also significant for colicin as compared to the amoxicillin (Figure 6).

The endogenous alkaline phosphatase (IAP) enzyme usually localizes to the apical brush border and participates in the de-phosphorylation of bacterial LPS in addition to the un-methylated cytosine-guanosine dinucleotides and flagellin, leading to reduced bacterial toxicity and inflammation responses (Vaishnava and Hooper, 2007).

In animals, the endogenous levels of IAP are reported to decrease at the weaning stage; hence pathogenic gram-negative bacteria (through an LPS-mediated mechanism) can upregulate inflammatory responses, leading to a symptomatic diarrhea. To address this issue, the use of exogenous IAP over-expression systems to modulate the animal’s overall IAP levels, promote gut health, and reduce the associated diarrhea has been suggested.

In a recent study, the effect of intestinal alkaline phosphatase (IAP) and sodium butyrate on LPS-induced intestinal inflammation was evaluated in pigs. The exogenous IAP was able to complement endogenous IAP levels and downregulate LPS-induced inflammatory responses *via* the RelA/p65 (NF-κB) route, demonstrating that such a treatment may indeed be beneficial in attenuating LPS-induced intestinal inflammation.

Colicin is a well-reported antimicrobial peptide that showed strong inhibition as compared to the repurposing antibiotics (Cascales et al., 2007; Jin et al., 2018). The study sequence of methyltransferase has shown a greater inhibitory potential. Recently, Ahmad et al. reported an antimicrobial peptide of 51 kDa from *Lysinibacillus*, with close sequence similarity to the methyltransferase (Ahmad et al., 2019). The interactions of methyltransferase, colicin, and amoxicillin were also studied with membrane protein of a gram-positive bacterial indicator, *Staphylococcus aureus* (Table 3 and Figure 7). The methyltransferase has also been observed to interact more significantly than colicin and amoxicillin. The interaction of colicin with *S. aureus* membrane protein was also observed to be significant, but it was less than methyltransferase. The antibiotic amoxicillin has also been observed significantly, but it was less than the interaction of methyltransferase. ent A-col E1, an antimicrobial peptide, was recently reported against *S. aureus* (Simons et al., 2020; Fathizadeh et al., 2020).

## Conclusion

Enzymes play a significant role for the expression of cellular proteins, cell wall polysaccharides, nucleic acids, and other cellular metabolites that are required for the survival of the cell. The use of enzymes as bacteriophage holins and their membrane-disrupting activity, anti-staphylococcal lytic enzymes, and membrane-targeted enzybiotics has recently been highlighted by much research. This *in silico* based study also explores the use of methyltransferase against gram-negative and gram-positive bacteria. The active sequences of this enzyme need to be explored. In this regard, we recommended the designing of short peptides using the methyltransferase sequence and evaluation of antimicrobial potential of these peptides that could be beneficial to develop a peptide-based enzymobiotic against gram-positive and gram-negative bacteria and pathogenic bacteria.

## Data Availability

The original contributions presented in the study are included in the article/Supplementary Material; further inquiries can be directed to the corresponding author.
